# Stonin 2 Overexpression is Correlated with Unfavorable Prognosis and Tumor Invasion in Epithelial Ovarian Cancer

**DOI:** 10.3390/ijms18081653

**Published:** 2017-07-29

**Authors:** Xiaoying Sun, Weijing Zhang, Han Li, Chunhao Niu, Yulan Ou, Libing Song, Yanna Zhang

**Affiliations:** State Key Laboratory of Oncology in South China, Collaborative Innovation Center for Cancer Medicine, Department of Gynecologic Oncology, Cancer Center, Sun Yat-Sen University, No. 651, Dongfeng Road East, Guangzhou 510060, China; sunxiaoy@sysucc.org.cn (X.S.); zhangwj@sysucc.org.cn (W.Z.); lihan@sysucc.org.cn (H.L.); niuchh@sysucc.org.cn (C.N.); ouyl@sysucc.org.cn (Y.O.); songlb@sysucc.org.cn (L.S.)

**Keywords:** *STON2*, Ovarian cancer, Prognosis, Biomarker, intraperitoneal metastasis, intraperitoneal recurrence, neoadjuvant chemotherapy, platinum resistance

## Abstract

Stonin 2 (*STON2*), which functions in adjusting endocytotic complexes, is probably involved in the monitoring of the internalization of dopamine D2 receptors which have an inhibitory action of dopamine on tumor progression. However, its clinical significance in tumor progression and prognosis remains unclear. We explored the association between *STON2* and the clinicopathological characteristics of epithelial ovarian cancer (EOC). The *STON2* levels in ovarian cancer and normal cell lines and tissues were detected by real-time PCR and Western blot analyses. *STON2* protein expression was also detected by an immunohistochemical analysis. The clinical significance of *STON2* expression in ovarian cancer was statistically analyzed. *STON2* significantly increased in the ovarian cancer cell lines and tissues compared to the normal ones. In the 89 *EOC* samples tested, *STON2* expression was significantly correlated with intraperitoneal metastasis, intestinal metastasis, intraperitoneal recurrence, ascites containing tumor cells, and CA153 level. Moreover, patients with *STON2* protein overexpression were more likely to exhibit platinum resistance and to have undergone neoadjuvant chemotherapy. Patients with high *STON2* protein expression had a tendency to have a shorter overall survival and a poor prognosis. A multivariate analysis showed that *STON2* was an independent prognostic predictor for EOC patients. In conclusion, *STON2* plays an important role in the progression and prognosis of ovarian carcinoma, especially in platinum resistance, intraperitoneal metastasis, and recurrence. *STON2* can be a novel antitumor drug target and biomarker which predicts an unfavorable prognosis for EOC patients.

## 1. Introduction

Ovarian cancer is the leading cause of death from gynecologic malignancy, and is associated with an incidence of approximately 22,280 new cases and an annual mortality rate of 14,240 deaths in the United States in 2016 [1]. Ovarian tumor treatments have been improving over the past few decades in particular, recent studies have explored platinum-containing chemotherapeutic agents as well as neoadjuvant chemotherapy (NAC) [2]. Regardless of these advances, ovarian cancer remains one of the deadliest types of gynecological carcinomas, of which the 5-year survival rate is merely 20–40%, primarily due to the occurrence of tumor metastasis and recurrence [3]. Due to the lack of specific symptoms and the absence of robust early diagnostic methods, epithelial ovarian cancer (EOC) is diagnosed in a majority of patients at an advanced stage (Federation of Gynecology and Obstetrics (FIGO) III or IV), leading to a disappointing prognosis [4,5]. On the other hand, patients with early-stage, localized ovarian cancer were reported to have an average five-year survival rate of 90% [6]. For decades, serum biomarkers such as CA125, HE4, and CA153 have been used to monitor the progress of ovarian cancer, and to detect the recurrence of ovarian cancer in a clinical setting. However, these biomarkers are neither extremely sensitive nor particularly specific for predicting cancer metastasis, recurrence, and prognosis, which may partly explain why their use has not significantly contributed towards improving the survival of EOC patients [7]. Therefore, further studies to identify reliable novel factors that can aid in the detection of tumor metastasis, predict tumor recurrence and survival, and provide personalized prediction for targeted therapy are essential in the prevention of tumor recurrence and for improving the prognosis of patients with EOC.

The *STON2* gene located on chromosome 14q in human encodes *Stonin 2*, which is a clathrin-related sorting protein that functions in adjusting endocytotic complexes. Moreover, many studies have suggested that *STON2* may play an important role in schizophrenia as an AP-2-dependent endocytic sorting adaptor for synaptotagmin internalization and recirculation [8,9,10,11,12]. More importantly, *STON2* probably participates in the monitoring of the internalization of dopamine 2 receptors D2 (D2Rs) [8,13]. It is well known that D2Rs play a significant role in the dopaminergic system and are responsible for the inhibitory action of dopamine on the stimulation of apoptosis, tumor progression, and the maturation of tumor microvessels. However, thus far, no research has specifically investigated the role of *STON2* on tumor progression and prognosis in ovarian cancer. Therefore, in this study we aimed to investigate the characteristics of *STON2* expression and its clinicopathological implications in ovarian cancer.

Here, we inspected *STON2* expression in ovarian cancer cell lines and tissues and in normal control cells and tissues. Correlations between many clinicopathological factors and survival in ovarian cancer, such as age, surgical stage, grade, lymph node metastasis, intraperitoneal metastasis, intraperitoneal recurrence, neoadjuvant chemotherapy, and platinum resistance, were analyzed by employing real-time PCR, Western blot analysis, immunohistochemistry, and statistical analyses.

## 2. Results

### 2.1. STON2 Expression is Higher in Ovarian Cancer Cell Lines than in Normal Ovarian Cell Lines

**Figure 1:**
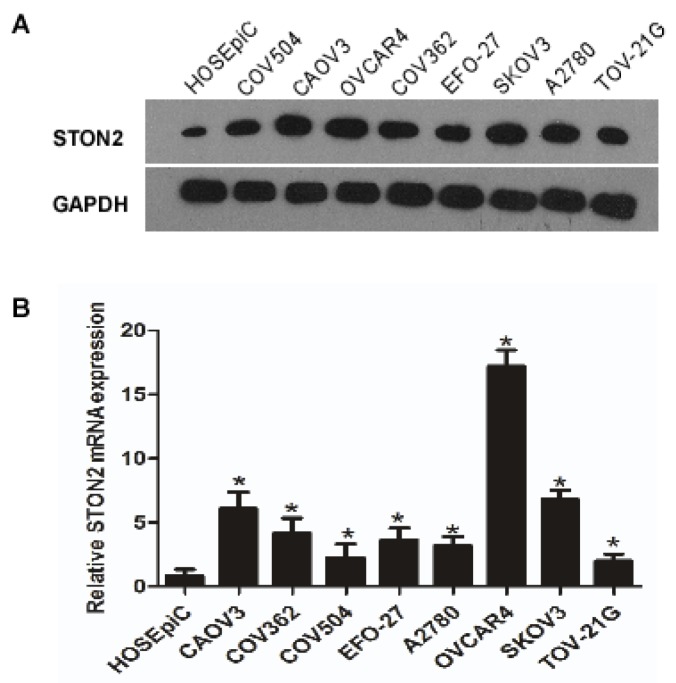
*STON2* mRNA and protein overexpression in ovarian cancer cell lines. The expression of *STON2* mRNA and protein in ovarian cancer and HOSEpiC cell lines was examined by Western blotting (**A**) and real-time PCR (**B**). The expression levels were normalized to the expression of *GAPDH*. Error bars: standard deviation (SD) of the mean from three parallel experiments. (* *p* < 0.05).

Real-time PCR and Western blot analyses were used to determine the expression of *STON2* mRNA and protein in ovarian cancer cell lines (CAOV3, COV362, COV504, EFO-27, A2780, OVCAR4, SKOV3, and TOV-21G) and in normal cells (HOSEpiC). The results of the real-time PCR and Western blot analyses revealed that all of the ovarian cancer cell lines overexpressed *STON2* protein and mRNA (Figure 1A,B).

### 2.2. STON2 Expression Significantly Increased in Ovarian Cancer Tissues Compared with Normal Control Tissues

**Figure 2:**
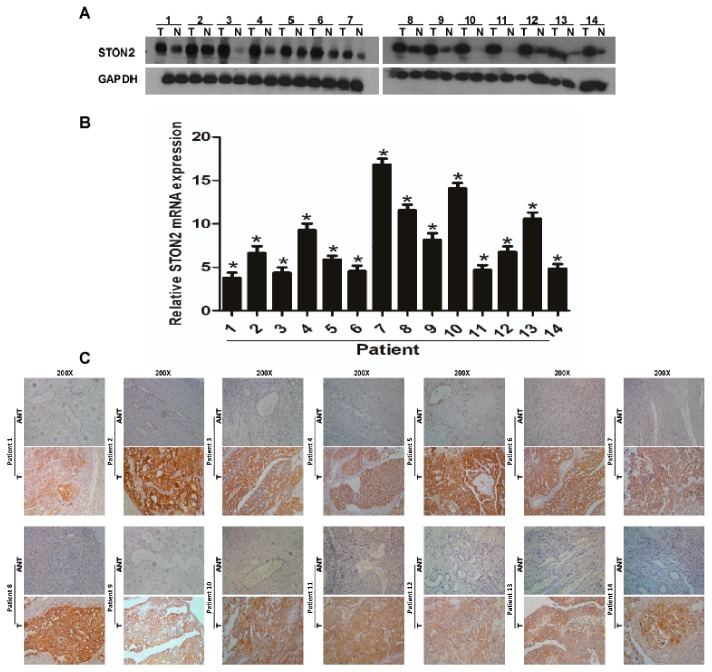
Overexpression of *STON2* mRNA and protein in ovarian cancer tissues. (**A**) Representative Western blots of *STON2* protein expression in 14 matched pairs of ovarian cancer (T) and adjacent noncancerous tissues (N). *GAPDH* was chosen as the loading control. (**B)** Average T/N ratios of *STON2* mRNA expression in paired ovarian cancer (T) and adjacent noncancerous tissues (N) were quantified by real-time PCR and normalized against the expression of *GAPDH*. Error bars, standard deviation (SD) of the mean calculated from three parallel experiments. (**C)** Immunohistochemical (IHC) assay of *STON2* protein expression in 14 pairs of matched ovarian cancer tissues (* *p* < 0.05).

We took advantage of real-time PCR, Western bloting, and immunohistochemical analyses to evaluate *STON2* expression in ovarian cancer and in normal control tissues at the mRNA and protein levels. As we predicted, the *STON2* mRNA and protein levels were obviously higher in most of the ovarian cancer tissues than in normal ovarian tissues (Figure 2A,B). Simultaneously, the results of the immunohistochemical staining also provided strong evidence that the *STON2* protein, which was intensively expressed in the cytoplasm, was upregulated in the ovarian cancer tissues compared to the normal ovarian tissues (Figure 2C).

### 2.3. STON2 Overexpression Was Related with the Clinical Features of Ovarian Cancer

**Figure 3:**
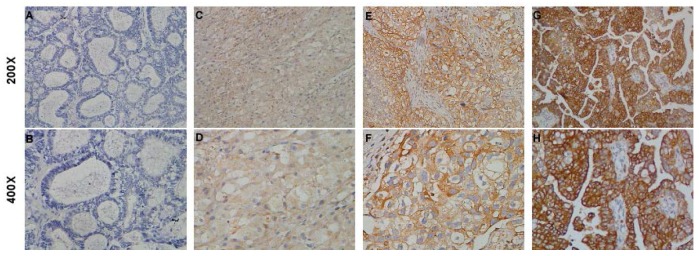
IHC detection of *STON2* expression in paraffin-embedded ovarian cancer tissues. Positive *STON2* staining was observed mainly in the cytoplasm. (**A**,**B**) *STON2* expression was not detected in adjacent noncancerous tissues; (**C**,**D**) representative images of weak *STON2* staining in ovarian cancer tissues; (**E**,**F**) representative images of moderate *STON2* staining in ovarian cancer tissues; (**G**,**H**) representative images of strong *STON2* staining in ovarian cancer tissues.

Considering the high expression of *STON2* in ovarian cancer, we further investigated its correlation with the clinical characteristics of ovarian cancer in 89 cases by immunohistochemistry. The percentages of patients with stages I, II, III, and IV tumors were 19.3%, 11.4%, 60.2%, and 9.1%, respectively. Among patients from 16 to 85 years of age, the median age was 50 years, and a total of 89 patients underwent the initial treatment including neoadjuvant therapy, surgery, or post-operation chemotherapy. According to the results of the immunohistochemical analysis, strong staining was detected in the cytoplasm in 44 of the 89 samples (49.4%, Table 1), indicating high *STON2* protein expression, while the remaining 45 EOC samples were poorly stained, indicating low *STON2* protein expression (50.6%, Table 1). Additionally, the mean optical density of *STON2* staining in the ovarian cancer samples was much higher than that in the normal control ovarian tissues (Figure 3). Furthermore, the results of the statistical analysis revealed a significant relationship between *STON2* expression and clinicopathological characteristics of ovarian cancer (Table 2), and a Spearman’s correlation analysis verified that high *STON2* expression was correlated with the following characteristics: intraperitoneal metastasis (*p* = 0.011), intestinal metastasis (*p* = 0.003), intraperitoneal recurrence (*p* = 0.006), ascites containing tumor cells (*p* = 0.016), and CA153 level (*p* = 0.041). Also, patients with *STON2* protein overexpression were more likely to exhibit platinum resistance (*p* = 0.033) and to have undergone neoadjuvant chemotherapy (*p* = 0.029). In contrast, *STON2* expression had no correlation with patient age; histological type; FIGO stage; differentiation grade; lymph node metastasis; serum CA125, CA199, CEA, NSE, and β-HCG levels; and other clinicopathological features (Table 3). Together, these results suggest that *STON2* may play an important role in disease development in ovarian cancer.

### 2.4. STON2 Overexpression Was Significantly Associated with a Poor Prognosis

**Figure 4:**
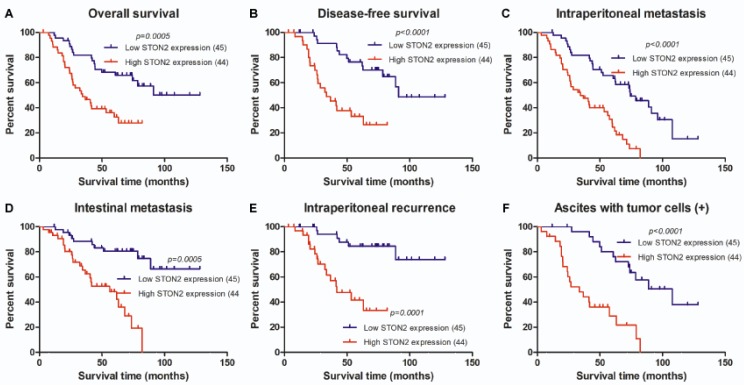

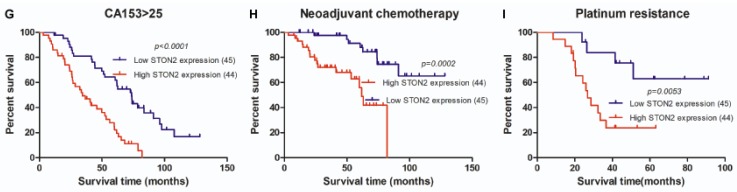
Kaplan–Meier curves of the univariate analysis data (log-rank test) showing the (**A**) overall survival (OS) and (**B**) disease-free survival (DFS) in patients with high versus low *STON2* expression. The Kaplan–Meier curves of the univariate analysis data (log-rank test) of patients with high versus low *STON2* expression. (**C**) OS of patients with intraperitoneal metastasis. (**D**) OS of patients with intestinal metastasis. (**E**) OS of patients with intraperitoneal recurrence. (**F**) OS of patients with ascites containing tumor cells (+). (**G**) OS of patients with CA153 > 25 U/mL. (**H**) OS of patients who received neoadjuvant chemotherapy. (**I**) OS of patients who exhibited platinum resistance.

A survival analysis revealed that the cumulative overall survival (OS) and disease-free survival (DFS) rates of ovarian cancer patients decreased with increase in *STON2* protein expression (Figure 4), indicating that *STON2* overexpression was associated with poor overall survival and survival with a high recurrence rate. In this study, patients with high *STON2* expression exhibited a median survival time of 34.36 months and a median progression-free survival time of only 5.1 months, while these values in patients with low *STON2* expression were 91.2 and 15.3 months, respectively. We also assessed the prognostic value of *STON2* expression in EOC patient subgroups stratified by serum biomarker levels such as CA153, CA199, intraperitoneal metastasis, ascites with tumor cells, neoadjuvant chemotherapy, intraperitoneal recurrence and so on (Figure 4). In a univariate Cox analysis, the *STON2* protein level (*p* < 0.001), CA153 (*p* = 0.001), CA199 (*p* = 0.009), intraperitoneal metastasis (*p* < 0.001), intestinal metastasis (*p* < 0.001), ascites with tumor cells (*p* = 0.001), neoadjuvant chemotherapy (*p* = 0.014), intraperitoneal recurrence (*p* < 0.001), and platinum resistance (*p* < 0.001) were significant prognostic factors (Table 4). In addition, multivariate Cox regression analysis further revealed that *STON2* protein expression level (*p* = 0.010), intraperitoneal recurrence (*p* = 0.011), and platinum resistance (*p* = 0.003) were indeed independent prognostic factors of ovarian cancer (Table 4). Taken together, these results suggest that as an independent prognostic factor, *STON2* may contribute to the prognosis of ovarian cancer.

## 3. Discussion

A growing body of evidence has indicated that Stonin 2 may have an important role in endocytosis by encoding a membrane protein that can sort clathrin-related proteins to internalize specific proteins. Furthermore, clathrin-mediated endocytosis represents a significant mechanism for recycling fully fused synaptic vesicles, and it is also involved in dopaminergic signaling, i.e., the attenuation of dopamine D2 receptors (D2Rs) [14,15]. Thus, Stonin 2 is probably involved in regulating the internalization of D2Rs [8,13]. Furthermore, ovarian cancer cells and endothelial cells all carry dopamine receptors, except ovarian cancer cells lacking dopamine receptor 3 (DR3) expression. Dopamine receptor 2 (DR2) is known to play an important role in the inhibitory functions of dopamine on microvessel density (MVD) and tumor growth as well as to have stimulatory functions on apoptosis. Via DR2, dopamine obstructs the vascular permeability factor/vascular endothelial growth factor-A (VPF/VEGF) or norepinephrine mediated invasion of ovarian cancer cells [16]. However, Stonin 2 participated in the internalization of D2Rs. After endocytosis, the D2Rs were shipped to the lysosomal pathway and degraded [17]. The attenuation of D2Rs abolished the dopamine-mediated inhibition of tumor cell invasion. Thus, *STON2* may directly or indirectly participate in the invasion of cancers, including ovarian cancer.

Despite the fact that the *STON2* protein plays an important role in endocytosis, there was little research investigating the relationship between *STON2* and tumors, particularly ovarian cancer, which still remains an important subject to be explored. Therefore, our study is a bold and creative attempt to preliminarily determine the impact of *STON2* expression on tumors, particularly in epithelial ovarian carcinoma.

This study has, for the first time, revealed that *STON2* protein expression was associated with the prognosis of ovarian cancer. Overexpression of *STON2* in patients with EOC was found to be significantly related with intraperitoneal metastasis, intestinal metastasis, intraperitoneal recurrence, ascites with tumor cells, and a high CA153 level. Moreover, patients with *STON2* overexpression were more likely to exhibit platinum resistance and to have undergone neoadjuvant chemotherapy. Also, further analysis of *STON2* expression showed that the survival time and progression-free survival time of epithelial ovarian carcinoma patients decreased with an increase in *STON2* expression. In the light of all the analyses, we concluded that *STON2* may be vital in the progression as well as the prognosis of ovarian cancer.

To the best of our knowledge, intraperitoneal metastasis and recurrence are the major risk factors that reduce survival in patients with ovarian cancer; moreover, these factors are of great importance in ovarian cancer staging [3]. In line with the International Federation of Gynecology and Obstetrics (FIGO) 2014, stage III has three substages: IIIA, IIIB, and IIIC. Stage IIIA is further divided into substage IIIA1, which means that patients only have positive retroperitoneal lymph nodes, and substage IIIA2, which means that the patients have microscopic intraperitoneal metastasis. Additionally, patients with macroscopic intraperitoneal metastasis but not parenchymal organ metastasis are usually categorized into stage IIIB or IIIC. The FIGO (2014) stated that patients with positive retroperitoneal lymph nodes alone commonly have a better prognosis than those with intraperitoneal metastasis. Thus, an early diagnosis of intraperitoneal metastasis is important for the survival of EOC patients [18]. However, only a few suitable tumor markers are currently used to predict intraperitoneal metastasis in the clinical setting. Due to all of these data, abnormal *STON2* expression was associated with intraperitoneal metastasis and recurrence, as well as a poor OS in ovarian cancer patients, suggesting that *STON2* overexpression is associated with a poor prognosis. Thus, *STON2* expression can serve as a predictor of intraperitoneal metastasis and recurrence in EOC patients and can be used to provide more precise cancer staging as well as guide a more radical personalized therapy by more effectively reducing the mortality rate of EOC patients.

Although surgery followed by postoperative chemotherapy has been the standard treatment for EOC for decades, patients with ovarian cancer still have a five-year survival rate of only approximately 30% [19]. One of the most significant prognostic factors for patients with advanced ovarian cancer is currently considered to be the capability to perform optimal cytoreduction [20]. If the conditions for optimal primary cytoreductive surgery are not present, neoadjuvant chemotherapy (NACT) is probably administered for patients with advanced ovarian cancer. Compared with primary cytoreductive surgery, neoadjuvant chemotherapy is associated with less blood loss, a superior percentage of optimal cytoreduction, as well as a more favorable quality of life [21]. Moreover, the National Comprehensive Cancer Network (NCCN) 2016 guidelines point out that advanced ovarian cancer patients especially with plenty of ascites and ascites cytology with malignant tumor cells, those with a clear histopathological diagnosis, and those that are evaluated by imaging or laparoscopy defining that the tumor would be troublesome to eliminate should undergo neoadjuvant chemotherapy. Additionally, we found that *STON2* overexpression indicated a tendency to have undergone neoadjuvant chemotherapy and the presence of ascites containing tumor cells, which is one of the indications for neoadjuvant chemotherapy. Therefore, *STON2* may be used as a NACT predictor for ovarian cancer patients to provide better guidance for individual treatment strategies, indicating that neoadjuvant chemotherapy may be more available for EOC patients with *STON2* overexpression.

Currently, ovarian cancer is lethal. Although chemotherapy is used to treat ovarian cancer, 20–30% of patients continue to exhibit platinum resistance [22]. In addition, platinum resistance is one of the most significant obstructions to the successful treatment of ovarian cancer. In our study, when EOC patients were found to have a high expression of *STON2*, they would more easily get platinum resistance and have a higher risk of worse survival outcomes. Our study is the first to identify the relationship between *STON2* and platinum resistance. Further studies with larger cohorts are needed to verify this association in EOC and study its underlying mechanism.

Recently, dopamine treatment has been considered as an effective and promising cancer therapy. Previous studies on mice transplanted with human breast, stomach, and colon tumors found that dopamine treatment could inhibit tumor angiogenesis and progression, as well as extend the life span in these animals [23,24]. Additionally, dopamine, via DR2, has been shown to prevent VPF/VEGF binding, the phosphorylation of receptors, subsequent signaling steps, and inhibit angiogenesis [25]. Moreover, dopamine can act via the D2R-cAMP signaling pathway to block the stimulatory effect of norepinephrine on tumor cell invasion [26]. A few reports have suggested that *STON2* may be involved in decreasing the expression of D2Rs by regulating their internalization [8,13]. Another study reported that dopamine treatment can increase tumor angiogenesis and progression on D2 receptor knockout mice [27]. Thus, *STON2* has potential for use as a therapeutic target to enhance the dopamine treatment’s efficacy in ovarian cancer.

## 4. Materials and Methods

### 4.1. Cell Lines

The ovarian cancer cell lines CAOV3, COV362, COV504, EFO-27, A2780, OVCAR4, SKOV3, and TOV-21G, and a normal ovarian epithelial cell line, HOSEpiC, were purchased from the American Type Culture Collection (ATCC, Manassas, VA, USA). All of the cells were grown in Dulbecco’s modified Eagle’s medium (Gibco, Grand Island, NY, USA) containing 10% fetal bovine serum (Invitrogen, Carlsbad, CA, USA), 100 U/mL penicillin, and 100 μg/mL streptomycin.

### 4.2. Tissue Specimens and Patient Information

A total of 89 paraffin-embedded ovarian cancer samples from 2002–2010 that had been pathologically confirmed at the Sun Yat-sen University Cancer Center were included in this study. In addition, freshly frozen ovarian cancer tissue samples and freshly frozen noncancerous ovarian biopsies were obtained from surgeries in the Sun Yat-sen University Cancer Center between May 2016 and January 2017 for research purposes. Table 1 summarizes the clinical information of all of the samples analyzed in this study. The follow-up time was between 5.1 and 126.5 months, and the median follow-up time was 49 months.

This trial was obtained approval from the Sun Yat-sen University Cancer Center Institutional Review Board (YB2016-064, 6 April 2016). Written informed consent was obtained from participants who provided the fresh tissue samples.

### 4.3. RNA Extraction and Real-Time PCR (RT-PCR)

The total RNA specimens from each specimen was extracted from the fresh tissues and cultured cells using the Trizol reagent (Invitrogen, Carlsbad, CA, USA) in line with the manufacturer’s instructions, and treated with RNase-free DNase. Then, cDNA was synthesized from 2 μg RNA obtained from each specimen using random hexamers in an iScript™ cDNA Synthesis Kit (Bio-Rad Laboratories, Hercules, CA, USA). The RT-PCR used was designedk by Primer Express Software Version 2.0 (Applied Biosystems, Foster City, CA, USA). Furthermore, the following primers were used for *STON2* and glyceraldehyde-3-phosphate dehydrogenase (*GAPDH*) amplification: *STON2* forward primer: 5’-GAGGATAAACCGACTGCCG-3’; *STON2* reverse primer: 5’-CGAGTTCAAGGTGGCAAAAG-3’; *GAPDH* forward primer: 5’-AATGAAGGGGTCATTGATGG-3’; *GAPDH* reverse primer: 5’-AAGGTGAAGGTCGGAGTCAA-3’.

### 4.4. Western Blot Analysis

Briefly, cells were washed thrice in ice-cold phosphate-buffered saline, lysed in 1× sodium dodecyl sulfate (SDS) lysis buffer (62.5 mmol/L Tris-HCl (pH 6.8), 10% glycerol, 5% 2-mercaptoethanol, and 2% SDS). The Bradford assay (Bio-Rad Laboratories, Hercules, CA, USA) was used to measure the protein concentrations. Fresh tissue samples were milled to powder in liquid nitrogen and lysed by SDS-PAGE sample buffer. The protein samples (30 μg) were separated on 7.5% SDS polyacrylamide gels, transferred to polyvinylidene fluoride (PVDF) membranes (Immobilon P, Millipore, Bedford, MA, USA), and blocked with 5% skimmed milk in Tris-buffered saline containing 0.1% Tween-20 (TBST) for 1 h at room temperature. After blocking, the membranes were incubated with anti-STON2 rabbit polyclonal antibodies (1:1000; Sigma, St. Louis, MO, USA; HPA003086) overnight at 4 °C. Then, the membranes were rinsed thrice with TBST for 10 min each time, and incubated with horseradish peroxidase-conjugated goat anti-rabbit IgG (1:2000; Santa Cruz Biotechnology, Dallas, TX, USA) for 1 h. Then, bound antibodies were detected using an enhanced chemiluminescence detection system ((Amersham Pharmacia Biotech, Piscataway, NJ, USA) according to the manufacturer’s instructions. GADPH (Santa Cruz Biotechnology) was chosen as the loading control.

### 4.5. Immunohistochemistry

The STON2 protein expression levels in the human ovarian cancer tissues were detected by immunohistochemical analysis. Briefly, 4 μm-thick paraffin-embedded sections were baked at 60 °C for 1 h, deparaffinized with xylene, rehydrated, and microwaved in EDTA antigen retrieval buffer. Next, high tension was used for antigen retrieval, and the specimens were treated with 3% hydrogen peroxide in methanol to quench endogenous peroxidase activity, followed by incubation with 1% bovine serum albumin to block nonspecific binding, and incubation with anti-rabbit STON2 polyclonal antibodies (1:1000; Sigma; HPA003086) at 4 °C overnight. Normal goat serum was used as the negative control. After washing, the tissue sections were treated with biotinylated anti-rabbit secondary antibody (Sigma), then incubated with streptavidin horseradish peroxidase complex (Sigma), immersed in 3-amino-9-ethyl carbazole. The sections were then counterstained with 10% Mayer’s hematoxylin, dehydrated, and mounted in Crystal Mount. Two researchers who knew nothing about the histopathological features and patient data of the sections evaluated the degree of immunostaining of each formalin-fixed, paraffin-embedded section. The score was due to both the proportion of positively stained tumor cells and the intensity of staining. The percentage of cancer cells was scored as follows: sections with <10% positive cancer cells were scored as 0; 10–50% positive cancer cells, 1; 50–75% positive cancer cells, 2; and tissue sections with >75% positive cancer cells, 3. The tissues were sorted into four grades based on staining intensity, as follows: 0 indicated no staining; 1 indicated weak staining (light yellow); 2 moderate staining (yellow brown); and 3 strong staining (brown). The staining index (0–9) was calculated as the product of the proportion of positive cells multiplied by the staining intensity score. The best cutoff value was defined as follows: a staining score of ≥6 was considered to have high STON2 expression, and a staining score of ≤4 indicated low STON2 expression.

### 4.6 Statistical Analyses

All of the statistical analyses were carried out with the statistical software package SPSS 20.0. The chi-square test and Fisher’s exact test were used to analyze the relationship between STON2 protein expression levels and clinicopathological characteristics. Additionally, bivariate correlations were computed by Spearman’s rank correlation coefficients. Patient survival was determined by a Kaplan–Meier analysis, and the differences were counted by the log-rank test. Cox’s proportional hazards regression model was applied to the multivariate analysis. A *p* value of < 0.05 in all of the analyses was considered statistically significant.

## 5. Conclusions

Overall, for the first time, the results of this study suggested that *STON2* is upregulated in ovarian cancer and its expression is correlated with intraperitoneal metastasis, intestinal metastasis, intraperitoneal recurrence, ascites with tumor cells, CA153 level, and poor prognosis in EOC patients. Additionally, patients in whom STON2 protein was overexpressed tended to be more likely to have undergone neoadjuvant chemotherapy and to exhibit platinum resistance. Therefore, STON2 might play an important role in the invasion of EOC, as well as serve as a novel cancer therapeutic drug target and biomarker that can predict an unfavorable prognosis in EOC patients.

## Figures and Tables

**Table 1:** Clinicopathological characteristics and tumor expression of *STON2* in epithelial ovarian cancer.

Characteristic	Number of Cases (%)
Age (years)	
≤50	35 (39.3)
>50	54 (60.7)
FIGO stage	
I	17 (19.3)
II	10 (11.4)
III	53 (60.2)
IV	8 (9.1)
Histological type	
Serous adenocarcinoma	69 (77.5)
Mucoid adenocarcinoma	15 (16.9)
Endometrial adenocarcinoma	3 (3.4)
Clear cell carcinoma	2 (2.2)
Lymph node metastasis	
Absent	29 (65.9)
Present	15 (34.1)
Intraperitoneal metastasis	
No	25 (28.1)
Yes	64 (71.9)
Intestinal metastasis	
No Yes	56 (62.9) 33 (37.1)
Expression of *STON2*	
Low or none High	45 (50.6) 44 (49.4)
Vital status (at last follow-up)	
Alive	42 (47.2)
Dead	47 (52.8)
Intraperitoneal recurrence	
No	48 (69.6)
Yes	21 (30.4)
Distant recurrence	
No	57 (82.6)
Yes	12 (17.4)
Residual tumor size (cm)	
≤1	75 (84.3)
>1	14 (15.7)
Differentiation grade	
G1/G2	13 (27.1)
G3	35 (72.9)
Neoadjuvant chemotherapy	
No	64 (71.9)
Yes	25 (28.1)
Postoperative chemotherapy	
No	7 (7.9)
Yes	82 (92.1)
HIPEC	
No	62 (71.3)
Yes	25 (28.7)
Ascites see tumor cells (+)	
No	21 (39.6)
Yes	32 (60.4)
Cytoreductive surgery	
No	12 (13.5)
Yes	77 (86.5)
Platinum resistance *	
No	14 (45.2)
Yes	17 (54.8)
CA125 (U/mL)	
≤35	5 (5.7)
>35	82 (94.3)
CA199 (U/mL)	
≤35	59 (67.0)
>35	29 (33.0)
CA153 (U/mL) ≤25 >25 NSE (U/mL) ≤15.2 >15.2 CEA (U/mL) ≤5.0 >5.0 β-HCG (U/mL) ≤3.0 >3.0	20 (23.3) 66 (76.7) 25 (37.3) 42 (62.7) 63 (81.8) 14 (18.2) 39 (78.0) 11 (22.0)
≤25	20 (23.3)
>25	66 (76.7)
NSE (U/mL) ≤15.2 >15.2 CEA (U/mL) ≤5.0 >5.0 β-HCG (U/mL) ≤3.0 >3.0	25 (37.3) 42 (62.7) 63 (81.8) 14 (18.2) 39 (78.0) 11 (22.0)

HIPEC, hyperthermic intraperitoneal chemotherapy; FIGO, International Federation of Gynecology and Obstetrics; * Epithelial ovarian carcinoma patients whose disease recurs in less than 6 months after initial platinum-based chemotherapy are termed platinum resistance. (Ovarian Cancer National Comprehensive Cancer Network (NCCN) 2017).

**Table 2:** Correlation between *STON2* expression and clinicopathological features of epithelial ovarian cancer.

Characteristic		Total	*STON2*		Chi-squared Test *p* Value	Fisher’s Exact Test *p* Value
			No or Weak Expression	Moderate or Strong Expression		
**Age (years)**	≤50	35	19 (21.3)	16 (18.0)	0.572	0.666
	>50	54	26 (29.2)	28 (31.5)		
**Histological type**	Serous adenocarcinoma	69	35 (39.3)	34 (38.2)	0.940	-
	Mucoid adenocarcinoma	15	8 (9.0)	7 (7.9)		
	Endometrial adenocarcinoma	3	1 (1.1)	2 (2.2)		
	Clear cell carcinoma	2	1 (1.1)	1 (1.1)		
**FIGO stage**	I	17	12 (13.6)	5 (5.7)	0.199	-
	II	10	3 (3.4)	7 (8.0)		
	III	53	25 (28.4)	28 (31.8)		
	IV	8	4 (4.5)	4 (4.5)		
**Lymph node metastasis**	Absent	29	19 (43.2)	10 (22.7)	0.431	0.521
	Present	15	8 (18.2)	7 (15.9)		
**Intraperitoneal metastasis**	No	25	18 (20.2)	7 (7.9)	0.011	0.018
	Yes	64	27 (30.3)	37 (41.6)		
**Intestinal metastasis**	No	56	35 (39.3)	21 (23.6)	0.003	0.004
	Yes	33	10 (11.2)	23 (25.8)		
**Vital status (at last follow-up)**	Alive Dead	42 47	27 (30.3) 18 (20.2)	15 (16.9) 29 (32.6)	0.014	0.020
**Intraperitoneal recurrence**	No Yes	48 21	31 (44.9) 17 (24.6)	6 (8.7) 15 (21.7)	0.006	0.008
**Distant recurrence**	No Yes	57 12	30 (43.5) 27 (39.1)	7 (10.1) 5 (7.2)	0.719	0.761
**Residual tumor size (cm)**	≤1	75	40 (44.9)	35 (39.3)	0.226	0.258
**Differentiation grade**	>1 G1/G2 G3	14 13 35	5 (5.6) 9 (18.8) 15 (31.2)	9 (10.1) 4 (8.3) 20 (41.7)	0.104	0.193
**Neoadjuvant chemotherapy** **Postoperative chemotherapy**	No Yes No Yes	64 25 7 82	37 (41.6) 8 (9.0) 3 (3.4) 42 (47.2)	27 (30.3) 17 (19.1) 4 (4.5) 40 (44.9)	0.029 0.671	0.035 0.714
**Platinum resistance**	No Yes	14 17	9 (29.0) 4 (12.9)	5 (16.1) 13 (41.9)	-	0.033
**HIPEC**	No	62	29 (33.3)	33 (37.9)	0.436	0.484
	Yes	25	14 (16.1)	11 (12.6)		
**Ascites with tumor cells (+)**	No	21	15 (28.3)	6 (11.3)	0.016	0.024
	Yes	32	12 (22.6)	20 (37.7)		
**Cytoreductive surgery**	No	12	7 (7.9)	5 (5.6)	0.563	0.484 0.758
	Yes	77	38 (42.7)	39 (43.8)		
**CA125 (U/mL)**	≤35 >35	5 82	4 (4.6) 39 (44.8)	1 (1.1) 43 (49.4)	0.159	0.202
**CA199 (U/mL)**	≤35	59	26 (29.5)	33 (37.5)	0.112	0.173
	>35	29	18 (20.5)	11 (12.5)		
**CA153 (U/mL)**	≤25 >25	20 66	14 (16.3) 29 (33.7)	6 (7.0) 37 (43.0)	0.041	0.072
**NSE (U/mL)**	≤15.2 >15.2	25 42	13 (19.4) 18 (26.9)	12 (17.9) 24 (35.8)	0.468	0.613
**CEA (U/mL)**	≤5.0 >5.0	63 14	28 (36.4) 9 (11.7)	35 (45.5) 5 (6.5)	0.179	0.240
**β-HCG (U/mL)**	≤3.0 >3.0	39 11	19 (38.0) 6 (12.0)	20 (40.0) 5 (10.0)	0.733	1.000

**Table 3:** Correlation between *STON2* expression and clinicopathological characteristics of epithelial ovarian cancer.

Variable	*STON2* Expression	
	Spearman’s Correlation Coefficient	*p* Value
Age	0.060	0.577
Histological type	0.011	0.915
FIGO stage	0.096	0.373
Intraperitoneal metastasis	0.268	0.011
Lymph node metastasis	0.119	0.443
Intestinal metastasis	0.311	0.003
Vital status (at last follow-up)	0.259	0.014
Intraperitoneal Recurrence	0.332	0.005
Distant Recurrence	−0.043	0.724
Residual tumor size (cm)	0.128	0.231
Differentiation grade	0.234	0.109
Neoadjuvant chemotherapy	0.232	0.029
Postoperative chemotherapy	−0.045	0.675
HIPEC	−0.08	0.442
Ascites with tumor cells (+) Cytoreductive surgery Platinum Resistance CA125 (U/mL) CA199 (U/mL) CA153 (U/mL) NSE (U/mL) CEA (U/mL) β-HCG (U/mL)	0.332 0.061 0.411 0.151 −0.169 0.220 0.089 −0.153 −0.048	0.015 0.568 0.022 0.163 0.115 0.042 0.475 0.184 0.739

**Table 4:** Cox regression univariate and multivariate analyses of prognostic factors in epithelial ovarian cancer.

Variable	Univariate Analysis			Multivariate Analysis		
	Number of Patients	*p*	Regression Coefficient (SE)	*p*	Relative Risk	95% Confidence Interval
***STON2***		0.001	2.834 (0.312)	0.001	37.631	4.794–295.37
Low expression	45					
High expression	44					
**Intraperitoneal metastasis**		0.000	9.351 (0.599)	0.358	0.355	0.039–3.238
No	25					
Yes	64					
**Intestinal metastasis**		0.000	3.048 (0.300)	0.603	1.556	0.294–8.246
No Yes	56 33					
**Intraperitoneal recurrence** No Yes	48 21	0.000	4.494 (0.354)	0.007	13.871	2.067–93.098
**Neoadjuvant chemotherapy** No Yes	64 25	0.016	2.060 (0.301)	0.234	2.744	0.520–14.473
**Ascites with tumor cells (+)** No Yes **Platinum Resistance** No Yes	21 77 14 17	0.002 0.000	4.711 (0.509) 5.694 (0.493)	0.896 0.004	1.196 24.220	0.082–17.515 2.794–209.925
**CA153 (U/mL)** ≤25 >25	20 66	0.004	4.628 (0.526)	0.074	0.113	0.010–1.235
